# TRENDS IN PREVALENCE OF HEARING LOSS AMONG OLDER ADULTS AND UNPAID FAMILY CAREGIVERS IN THE UNITED STATES: 2011–2022

**DOI:** 10.1093/geroni/igae098.2533

**Published:** 2024-12-31

**Authors:** Wuyang Zhang, Danielle Powell, Nicholas Reed

**Affiliations:** Johns Hopkins University, Baltimore, Maryland, United States; University of Maryland, College Park, Maryland, United States; Johns Hopkins University, Baltimore, Maryland, United States

## Abstract

Hearing loss (HL) prevalence rises with age and is a potential barrier to communication, a key component of caregiving relationships. With a rapidly aging population, the United States faces a surge in age-related HL prevalence in tandem with demand for disability-related care needs. We aim to estimate the trends in national prevalence of HL among (1) older adults receiving unpaid family care and (2) their caregivers. The National Health and Aging Trends Study (NHATS) and linked National Study of Caregiving (NSOC) provide nationally representative samples of Medicare beneficiaries and their family caregivers between 2011-2022. HL was measured through self-reported functional hearing ability (subjective) in both NHATS and NSOC until 2021, when NHATS introduced objective measures of hearing. In 2011, the prevalence of self-report HL was 31.7% (95% CI: 28.3%-35.4%) among care recipients and 7.9% (95% CI: 6.7%-9.3%) among caregivers. By 2022, the prevalence reached 34.9% (95% CI: 31.5%-38.4%) and 9.7% (95% CI: 8.1%-11.6%), respectively. Notably, prevalence of objective HL among care recipients more than doubled its subjective counterpart (73.5%; 95% CI: 69.2%-77.3%). Among caregiving dyads, 3.6% (95% CI: 3.5%-5.0%) had both parties reporting subjective HL and 5.7% (95% CI: 4.4%-7.3%) had caregivers with subjective HL assisting older adults with objective HL. Stratified analyses by sociodemographic characteristics suggested the prevalence of HL was higher among male, non-Hispanic White, or Hispanic care recipients along with spousal caregivers. Our findings suggest that HL is highly prevalent among both older care recipients and caregivers, and further investigation is needed to understand its caregiving consequences.

